# EMBRACE: One Small Story in Lupus—One Giant Challenge in Clinical Trials

**DOI:** 10.1002/acr2.11477

**Published:** 2022-06-24

**Authors:** Saira Z. Sheikh, Tessa R. Englund, Susan W. Burriss, Jonca Bull, Anya Harry, James G. Groark, Ashley M. Hall, Michelle Miller, David A. Roth

**Affiliations:** ^1^ University of North Carolina at Chapel Hill School of Medicine Chapel Hill North Carolina USA; ^2^ GlaxoSmithKline Collegeville Pennsylvania USA; ^3^ Strategic Regulatory Consultants Washington DC USA

## Abstract

Clinical trials of novel therapeutics in the United States have not been adequately representative of diverse populations, particularly racial and ethnic minorities. The challenges and consequences of underrepresentation in clinical trial recruitment are exemplified by the case of belimumab, a biologic treatment for systemic lupus erythematosus (SLE), a disease that is more prevalent in patients of Black African ancestry and of Hispanic/Latino ethnicity than in other patient populations. Although belimumab was found to be effective in phase 2 and 3 clinical trials in the general population, post hoc analyses of efficacy data in patients of Black African ancestry showed inconsistent results. Consequently, a cautionary statement regarding belimumab use in this population was added to the product label. To alleviate concerns that belimumab may not be safe and effective for patients of Black African ancestry, the Efficacy and Safety of Belimumab in Black Race Patients with SLE (EMBRACE) study was conducted in a post‐marketing commitment to the Food and Drug Administration. The study recruited only patients who self‐identified as being of Black race; its findings led to the removal of the cautionary labeling of belimumab use in patients of Black African ancestry. Our manuscript highlights the critical lessons learned from the successes and failures of the EMBRACE study. It also provides suggestions for overcoming health disparities, highlighting strategies for conducting well‐designed clinical trials to overcome systematic barriers to diversity in recruitment, with a focus on enacting long‐term support to ensure equity in the process, products, and benefits from drug development and clinical trials.

Clinical trials of novel therapeutics in the United States have not been adequately representative of diverse populations, particularly racial and ethnic minorities. Underrepresentation of minority participants in clinical trials leading to Food and Drug Administration (FDA) approval of new drugs is a persistent issue, especially with respect to patients of Black African ancestry, who are consistently underrepresented in clinical trials based on the US population and targeted disease prevalence (1).

Systemic lupus erythematosus (SLE) is a chronic, inflammatory autoimmune disease that primarily affects women of childbearing age and disproportionately affects individuals of Black African ancestry and of Hispanic/Latino ethnicity, who have greater morbidity and mortality from the disease. During 2000‐2015, SLE was the fifth leading cause of death among Black/African American and Hispanic/Latino women 15‐24 years of age (2).

Despite greater prevalence among racial and ethnic minorities, marked gaps exist between populations affected by SLE and those enrolled in clinical trials. White patients constitute 33% of prevalent SLE cases but are overrepresented in SLE randomized controlled trials (RCTs), representing 51% of participants. In contrast, Black/African American patients make up 43% of prevalent SLE cases but are underrepresented in SLE clinical trials, comprising only 14% of RCT participants (3). There are a myriad of patient‐ and provider‐side barriers to overcome in order to improve racial and ethnic representation in clinical trials. These include, but are not limited to, patients’ awareness about clinical trial opportunities, lack of access to referring providers, logistical challenges, and a lack of trust in clinical research and the health care system (1, 4). On the provider side, treating physicians’ lack of access to clinical trial information and effective referral partnerships with clinical trial sites, and attitudes/implicit biases, limit referrals to clinical trials (1). All the patient‐ and provider‐side barriers exist at different levels in the past and present context of structural racism and discrimination, both in and out of the health care and research continuum (5).

The challenges and consequences of underrepresentation in clinical trial recruitment are exemplified in the case of belimumab, the first drug approved for SLE in over 50 years. In phase 2 and 3 RCTs, belimumab was efficacious in the general trial population. However, owing to broader challenges with multinational recruitment of diverse patients, the number of patients of Black African ancestry included in the belimumab trials was small (4%‐14% of patients), and post hoc exploratory subgroup analyses revealed inconsistent results regarding belimumab's efficacy in this population. A consequence of these exploratory analyses requested by the FDA was that the product label advised health care practitioners to exercise caution when considering belimumab for Black/African American patients: “Although no definitive conclusions can be drawn from these subgroup analyses, caution should be used when considering BENLYSTA treatment in Black/African American patients with SLE.” These warnings led to confusion about prescribing belimumab to patients of Black race/African American descent, likely resulting in delayed access to treatment for many patients with SLE who incur the greatest disease burden.

The cautionary labeling may have implied that Black/African American race alone was associated with differences in treatment response. Race is a social construct, defined by the Oxford English Dictionary as “…any of the (putative) major groupings of mankind, usually defined in terms of distinct physical features or shared ethnicity, and sometimes (more controversially) considered to encompass common biological or genetic characteristics.” SLE‐treatment responses have been shown to differ in some cases by ancestry (6). As with many conditions though, the relative contributions of genetics; bias; discrimination; environmental, socioeconomic, and cultural factors; and access to care in racial and ethnic differences in treatment response and disease outcomes are not well understood (7).

To alleviate concerns that belimumab may not be safe and effective for patients of Black African ancestry, GlaxoSmithKline, the sponsor, agreed to conduct an RCT, the Efficacy and Safety of Belimumab in Black Race Patients with SLE (EMBRACE) study (NCT01632241) (8), as a post‐marketing commitment to the FDA. Although EMBRACE was born from a failure of previous RCTs to enroll diverse and representative participants, it represents a major success in enrolling a well‐defined minority patient population into a large RCT. The successes and failures that led to the FDA's request to conduct this post‐marketing commitment trial, and those resulting from it, highlight critical lessons learned that can inform future well‐designed clinical trials with adequate patient representation. EMBRACE concluded after years of intensive, multinational efforts and extensive resources leveraged to enroll self‐identified Black patients with SLE into the trial. Although the study did not meet the primary endpoint, it provided enough evidence of efficacy of belimumab for the FDA to remove the cautionary statement from the product label. Table 1 highlights critical lessons from EMBRACE, discussed subsequently.

**Table 1:** EMBRACE lessons learned and strategies to inform the design and conduct of future clinical trials

Strategy	The EMBRACE experience	Effective strategies to inform future clinical trials
Enrollment targets	EMBRACE had an enrollment target of 816 patients over 3.5 years (March 2013‐September 2016), which was predicted to be 50% slower than the phase 3 belimumab trials, which had no specific demographic targets. By November 2014, recruitment tracked at 137 (30% of the projected recruitment goal at that time point). The Food and Drug Administration subsequently agreed to decrease the enrollment target to 500, which was met with 503 patients being enrolled in 3.5 years. Ultimately, the study was underpowered and did not reach statistical significance.	Recommendations include the use of adaptive enrollment and retention practices, community outreach, and use of expanded access programs. The use of electronic health record data and disease registries can facilitate identification of sites and/or patients who may meet eligibility criteria for a clinical trial. There have since been expert‐informed recommendations for effective and efficient recruitment planning (18), as well as guidance to increase diversity in clinical research (19) that include practical steps that can support realistic recruitment and enrollment milestones, such as using historic and benchmarked data to estimate enrollment timelines.
Inclusion criteria and study design	Patients with SLE were eligible if they self‐identified as being of Black race, had active disease (without lupus nephritis) with higher disease activity scores than observed in previous trials, and were on stable doses of medications. Stable medications may be a challenging criterion to meet for patients with high disease activity. The stringent eligibility criteria resulted in a higher than anticipated screening failure rate (45% rather than 35%) and deterred widespread screening at the registered sites. Because belimumab was already approved in various markets, enrolling patients into a placebo‐controlled study for a marketed product was considered unethical by some physicians and cited as a reason for not referring patients into the trial, thereby reducing the number of potential study sites.	Recommendations include the simplification and broadening of eligibility criteria and real‐world pragmatic study designs. Sponsors can attempt to use adaptive clinical trial approaches such as umbrella, basket, and platform designs to study multiple targeted therapies or disease subtypes in the context of a single clinical trial protocol (20).
Infrastructure development and site selection	It was anticipated that 160 sites would participate in EMBRACE to achieve a target sample of 816 participants. Of the 950 sites identified and contacted, only 240 sites expressed interest, and 114 were selected. Of the 114 selected, only 64 sites recruited at least one patient into the study. Despite extensive global outreach and feasibility efforts, sites from only seven countries were selected for EMBRACE owing to a multitude of factors, including a lack of sufficient clinical trial infrastructure and qualified personnel, noncomparable standards of medical care, security concerns, and small potential pool of eligible patients with SLE.	Although the limitations of country‐level restrictions may be difficult to overcome, the need for balancing rigorous trial design, patient selection criteria, and competition from concurrent clinical trials should be carefully weighed against recruitment challenges in future trials. Master protocols for clinical trial designs offer opportunities to eliminate competition with other trials by using one overarching protocol designed to answer multiple questions, particularly when sponsors are attempting to enroll difficult‐to‐recruit populations (20).
Competition from other clinical trials and extraneous circumstances	During the time EMBRACE was conducted, three concurrent belimumab SLE trials were also actively recruiting patients, in addition to competitor trials for other novel lupus therapeutics. The Ebola crisis, which started in 2013, created unforeseen challenges in site selection of countries in West Africa. Additionally, racial demographics in certain countries and regions (such as Asia‐Pacific countries and many European countries) and/or negative social views to self‐report as being of Black African ancestry (or mixed race) were not conducive to recruitment of patients into a race‐focused trial in some countries.	Severe unforeseen disruptions, such as the coronavirus disease 2019 pandemic, present strategic opportunities to re‐envision clinical trial infrastructure with respect to the conduct of patient‐centered and direct‐to‐patient clinical trials, with use of innovative technology potentially using virtual visits and decentralized clinical trials.
Subgroup analyses and randomization	If patients of Black African ancestry had been adequately represented and randomized in the phase 2 and 3 trials of belimumab, with prespecified subgroup analyses that were adequately powered, the EMBRACE study likely would not have been necessary.	Recommendations include recruiting and enrolling participants representative of the patient population characteristics. Subgroup analyses should be prespecified and adequately powered if results will be used in labeling/treatment indications. Sponsors should communicate with regulatory agencies prior to trial initiation to ensure that enrollment targets adequately represent populations of interest. Prespecified subgroup analyses and plans for how to interpret unforeseen or underpowered analyses should be in place. Also, sponsors should provide transparent data and interpretation of results from clinical trials, with limitations clearly outlined.
Educate site coordinators and maintain site engagement	To encourage patient recruitment, calls and site visits from the sponsor were frequent with regular communication about study progress (eg, encouraging healthy competition among sites). Site monitors and coordinators were provided with study toolkits to educate patients and families about the clinical trial process and assist sites in raising awareness of the study through local outreach (eg, digital and print promotions). Also, research sites were educated on how to effectively communicate study objectives and demographic requirements to potential participants, and how to talk openly to all patients about their racial background regardless of whether they did or did not appear to have Black African ancestry.	Consistent communication with sites is critical to maintain site engagement, support progress toward recruitment targets, and address and improve unique study characteristics that may pose a challenge to site recruitment. Sites should be equipped with tailored, culturally competent patient‐facing study‐related toolkits, designed in accordance with health‐literacy practices and made accessible in a variety of engaging formats and languages. Sponsor‐led clinical trial educational programs can provide ongoing practical training and educational support that are tailored to the needs of each site and that address key barriers to patient recruitment.
Focus on local outreach, education, and engagement to facilitate buy‐in and build trust in clinical research	To assist community education and outreach, resources with contact information for local patient advocacy groups were provided to study sites. Local opportunities for outreach, such as lupus annual meetings, lupus walks and outings, workshops and seminars, and community health fairs, were highlighted to participating sites. All participating US sites were provided with a list of such events being held in their community. Research sites were also recognized for their participation in various awareness campaigns. National study awareness campaigns were costly with low yield and were not perceived to be as effective as partnerships with local patient‐facing organizations.	Local partnerships and outreach may be more feasible than national campaigns, with approaches tailored to the local community to increase awareness and recruitment of patients into clinical trials. Formal training is needed for investigators and referring clinicians to use effective, patient‐centered communication techniques that build trust and strengthen relationships with underrepresented patients. This is an area of growing interest and a clear opportunity for sponsors and other stakeholders to mobilize resources to develop training programs specifically to improve patient–clinician communication around lupus clinical trials.
Provide patient support to participate in trials	Patient travel and transportation were frequently cited as a limitation to patient recruitment in EMBRACE. To help overcome barriers to participation, patients were provided with practical support, including help with transportation to and from the study site, and overnight stays for patients who lived far from study centers. Working with study sites to establish a reliable transportation service early on was a key to retention at non‐community‐based sites.	Sponsors should establish transportation, childcare, and other logistical support services for patients as early as possible. Practical measures include use of flexible and adaptive study visits through initiatives such as mobile research centers and telemedicine research visits, which may improve patient accessibility and reduce conflicts with work or family commitments. Another recommendation is to provide sponsorship and support to create clinical trial infrastructure and train investigators in the communities where patients reside to minimize travel requirements.

Abbreviations: EMBRACE, Efficacy and Safety of Belimumab in Black Race Patients with SLE; SLE, systemic lupus erythematosus.

It is important to consider and strive toward three key elements when designing and recruiting for future clinical trials: 1) study participants should reflect the diversity of the patient population affected by the disease; 2) systematic reform and clinical trial infrastructure at the sponsor level should be developed to establish sustainable recruitment platforms and partnerships; and 3) clinical trials should be powered adequately and ideally designed with prespecified subgroup analyses relevant for the proposed treatment and population. In phase 2 and 3 RCTs of belimumab, participants of Black African ancestry were underrepresented based on the prevalence of SLE in the United States. If the participants in these studies had adequately reflected the SLE population, the EMBRACE study might not have been necessary. The FDA has since released guidance to enroll individuals “who reflect the characteristics of clinically relevant populations with regard to age, sex, race, and ethnicity,” highlighting that inadequate participation and/or analyses of data from clinically relevant populations can lead to insufficient safety and efficacy for product labeling (9). It is imperative that sponsors of future trials communicate with regulatory agencies to ensure that, if warranted, prespecified subgroup analyses are appropriately planned to mitigate the risk of Type I error that may lead to potentially harmful erroneous findings about efficacy in subgroups. The success of this strategy is highlighted in the Belimumab International Study in Lupus Nephritis (BLISS‐LN) study (NCT01639339), in which randomization was stratified according to race (Black vs. non‐Black), allowing for meaningful conclusions about efficacy of the treatment.

Guidance, transparency, and pretrial discussions and consensus with regulatory bodies are needed to facilitate development of appropriate study designs and subgroup analyses to identify and overcome avoidable barriers to bring a safe and effective drug to market. This is particularly important for drugs developed for diseases more prevalent among racially and ethnically diverse populations and other underrepresented groups. Sponsors should not be deterred from developing such drugs because of ambiguity, risk of unsupported labeling, and costly post‐marketing requirements, which can be avoided through formative discussions and due diligence before initiating clinical trials. The FDA recently released draft guidance for sponsors on developing a Race and Ethnicity Diversity Plan to identify and enroll adequate numbers of participants from underrepresented populations into clinical trials (10). This guidance includes several recommendations for sponsors to improve racial and ethnic representation in clinical trials that could address many of these uncertainties in the drug development process. Assessment of racial, ethnic, or other relevant covariate characteristics with potential to affect pharmacodynamics and pharmacokinetics will be useful, as recommended in the Race and Ethnicity Diversity Plan guidance (10), and also in post‐marketing surveillance to understand treatment response as well as long‐term outcomes in diverse populations.

Unsubstantiated cautionary labeling may further contribute to health disparities with avoidable and potentially harmful claims that continue to unintentionally perpetuate the idea that, at times, race can be treated as a biological characteristic rather than a socially derived construct. The cautionary statement for use of belimumab in patients of Black African ancestry remained on the product label for almost 9 years until it was removed following completion of the EMBRACE trial. The decision to use results from post hoc subgroup analyses to justify cautionary labeling exemplifies the real consequences of health inequities stemming from race‐based categories. Consequently, access to belimumab may have been delayed or denied for patients of Black African ancestry, compounding disparities in the disproportionate burden of SLE that these patients face.

The EMBRACE trial encountered unforeseen global challenges (eg, the Ebola crisis, which started in 2013, see Table 1) and delays in patient recruitment that may have affected the study's outcomes. These challenges also necessitated the development and implementation of a range of strategies (see Table 1) that were effective at improving patient recruitment and addressing barriers to enrolling a specific patient population, which ultimately facilitated the approval of belimumab indication labeling for patients of Black African ancestry. These strategies included working locally to encourage collaboration between health care practitioners and their local advocacy groups, engaging in community outreach, and raising the visibility of local study sites (see Table 1).

Beyond enhancing recruitment approaches and infrastructure, there are many opportunities to improve the participant experience, and subsequently improve representation and retention in clinical trials for novel lupus therapeutics. Patients face a difficult journey in obtaining a lupus diagnosis (11, 12), and then the continued stressors and day‐to‐day challenges of living with this chronic disease. Lupus disproportionately affects women of childbearing age (13). Difficulties and uncertainties arising from the onset and management of lupus often coincide with major life events as well as personal and professional aspirations, including, but not limited to, reproductive goals (13) and career potential (14). The magnitude of impact that lupus has on quality of life underscores the need to incorporate diverse patients’ perspectives throughout the research process, from the development of research questions to the selection of endpoints and patient‐reported outcomes that are meaningful to patients (15, 16). The use of patient‐reported outcomes in clinical trials can help clinicians, investigators, and sponsors to better understand the unique experiences and concerns of underrepresented patients, which extend beyond disease activity and organ damage outcomes often prioritized by clinicians (17).

Sponsors and researchers can and should address health disparities by working to overcome systematic barriers to diversity in clinical trials and enacting long‐term support to ensure equity in the process, products, and benefits from drug development and clinical trials. We believe there is a tremendous need and opportunity to develop and conduct well‐designed clinical trials that expand the use and quality of treatments available for diverse patient populations, irrespective of disease state. Sponsors, regulatory agencies, and researchers can act to design well‐represented, diverse, and robust clinical trials that ensure equitable access and benefit to all patients.

## AUTHOR CONTRIBUTIONS

All authors were involved in drafting the article or revising it critically for important intellectual content, and all authors approved the final version to be published.

## ROLE OF THE STUDY SPONSOR

GlaxoSmithKline supported the authors in the development of this editorial, including study design, data collection, data analysis, interpretation of the data, and the editing of the manuscript. GlaxoSmithKline was involved in the decision to submit the manuscript for publication.

## Supporting information


**Disclosure Form**:  Click here for additional data file.
